# We Welcome Findings from Further Afield

**DOI:** 10.13023/jah.0501.01

**Published:** 2023-04-01

**Authors:** Randy Wykoff, Rachel E. Dixon

**Affiliations:** East Tennessee State University, wykoff@etsu.edu; Journal of Appalachian Health, managingeditor.jah@gmail.com

**Keywords:** Appalachia, academic research, submissions, interdisciplinary research

## Abstract

For the past five years, the *Journal of Appalachian Health* has published timely, high-quality research from within Appalachia. We also welcome submissions from those working outside of Appalachia who produce quality research of direct relevance to our region.

The two of us are honored to take over the editorial leadership of the *Journal of Appalachian Health*. Dough Scutchfield, as Editor-in-Chief, and Charlotte Seidman, as Managing Editor, not only created and launched the Journal, but also left enormous shoes to fill.

We have made a few key observations about the Journal as it enters its fifth year. The first is that it is obviously filling an important and unmet need. The health challenges of the people of Appalachia are well recognized; yet prior to the launch of the Journal, there was no peer-reviewed research publication focused solely on these issues in an Appalachian context. The second observation is that there is tremendously important research being conducted across the region. In the last four issues alone, authors have come from 30 different universities and organizations (at community, state and federal levels). Topics covered have been as diverse as the authorship, ranging from diabetes preventive care^1^–^4^ and diseases of despair^5^–^7^ to community leadership^8^–^12^ and multi-sector partnerships and initiatives.^13^–^15^ The third observation of note is that what is being published is of interest to readers—there have been almost 5,000 article downloads in the past year alone. Additionally, over half of recent downloads have come from outside of Appalachia, including from a dozen foreign countries.

These observations, of course, are highly reassuring; they motivate all of us associated with the Journal to “keep on keeping on.” Beyond this, they raise an important question: If the research coming out of Appalachia is of interest to so many people *beyond* the region, might research conducted further afield be of interest to our readers here? Other organizations associated with Appalachia have recognized this opportunity. The *Journal of Appalachian Studies*, for example, “invites scholarship which compares the Appalachian region to other regions in the world and places the region in a critical, global context.”^1^ Given the universality of many of the health challenges that face the people of Appalachia, quality research on issues of regional importance could have a place in the Journal, even if the work was carried out elsewhere.

We extend a warm welcome, then, to researchers from outside the region to submit their work when it is of direct relevance to the people of Appalachia. In this vein, quality research on inter-generational poverty, opioid misuse, the challenges of an aging rural population, and many other related issues is of tremendous interest to all of us working in Appalachia. Just as work conducted in Appalachia will continue to be published outside of the *Journal of Appalachian Health*, we hope that research conducted elsewhere, which could inform—and even transform—our practices in the region, will be submitted and published within these pages.
